# Bis[4,4,5,5-tetra­methyl-2-(pyridin-2-yl-κ^2^ *N*)imidazoline-1-oxyl 3-oxide-κ*O*]tris­(nitrato-κ^2^ *O*,*O*′)terbium(III)

**DOI:** 10.1107/S1600536812040287

**Published:** 2012-10-10

**Authors:** Dong-jiao Li

**Affiliations:** a Chemical Institute, Linyi University, Linyi Shandong 276005, People’s Republic of China

## Abstract

The title compound, [Tb(NO_3_)_3_(C_12_H_16_N_3_O_2_)_2_], was prepared from the nitroxide radical ligand 4,4,5,5-tetra­methyl­-2-(pyridin-2-yl)-imidazoline-1-oxyl-3-oxide and Tb^III^ nitrate. The Tb^III^ ion adopts a doubly-capped square-anti­prismatic coord­ination environment defined by three chelating nitrate anions and two *N*,*O*-bidentate nitronyl nitroxide radical ligands. Weak C—H⋯O hydrogen bonds connect the molecules into a three-dimensional framework. The title structure is isotypic with the Ho analogue [Li (2012 ▶). *Acta Cryst.* E**68**, 550].

## Related literature

For background to the use of rare earth complexes with nitroxide radicals in coordination chemistry, see: Sutter *et al.* (1998 ▶); Kahn *et al.* (2000 ▶); Lescop *et al.* (2000 ▶). For the structures of related complexes, see: Li *et al.* (2004*a* ▶,*b* ▶, 2005 ▶); Li (2012 ▶).
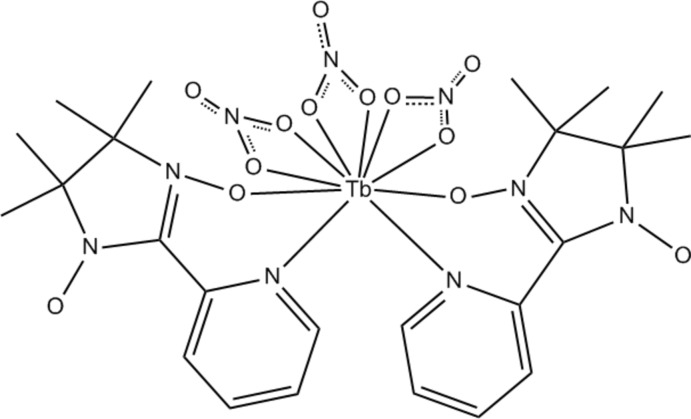


## Experimental

### 

#### Crystal data

[Tb(NO_3_)_3_(C_12_H_16_N_3_O_2_)_2_]
*M* *_r_* = 813.51
Monoclinic, 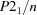

*a* = 12.292 (3) Å
*b* = 11.114 (2) Å
*c* = 23.264 (5) Å
β = 98.37 (3)°
*V* = 3144.6 (11) Å^3^
*Z* = 4
Mo *K*α radiation
μ = 2.33 mm^−1^
*T* = 293 K
0.20 × 0.20 × 0.20 mm

#### Data collection

Rigaku Saturn CCD area-detector diffractometer
Absorption correction: multi-scan (*SADABS*; Sheldrick, 2004 ▶) *T*_min_ = 0.581, *T*_max_ = 1.000
25443 measured reflections
5554 independent reflections
4726 reflections with *I* > 2σ(*I*)
*R*_int_ = 0.059

#### Refinement

*R*[*F* ^2^ > 2σ(*F* ^2^)] = 0.031
*wR*(*F* ^2^) = 0.071
*S* = 1.01
5554 reflections
432 parameters
H-atom parameters constrained
Δρ_max_ = 0.89 e Å^−3^
Δρ_min_ = −0.95 e Å^−3^

### 

Data collection: *CrystalClear* (Rigaku, 2008 ▶); cell refinement: *CrystalClear*; data reduction: *CrystalClear*; program(s) used to solve structure: *SHELXS97* (Sheldrick, 2008 ▶); program(s) used to refine structure: *SHELXL97* (Sheldrick, 2008 ▶); molecular graphics: *SHELXTL* (Sheldrick, 2008 ▶); software used to prepare material for publication: *SHELXTL*.

## Supplementary Material

Crystal structure: contains datablock(s) I, global. DOI: 10.1107/S1600536812040287/mw2078sup1.cif

Structure factors: contains datablock(s) I. DOI: 10.1107/S1600536812040287/mw2078Isup2.hkl

Additional supplementary materials:  crystallographic information; 3D view; checkCIF report

## Figures and Tables

**Table 1:** Hydrogen-bond geometry (Å, °)

*D*—H⋯*A*	*D*—H	H⋯*A*	*D*⋯*A*	*D*—H⋯*A*
C22—H22⋯O13^i^	0.93	2.55	3.440 (5)	161
C17—H17*B*⋯O6^ii^	0.96	2.40	3.327 (5)	161
C6—H6*B*⋯O3^iii^	0.96	2.55	3.473 (5)	161
C24—H24⋯O9^iv^	0.93	2.38	3.211 (5)	148

Symmetry codes: (i) 
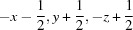
; (ii) 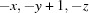; (iii) 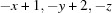; (iv) 
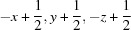
.

**Fig. 1.**
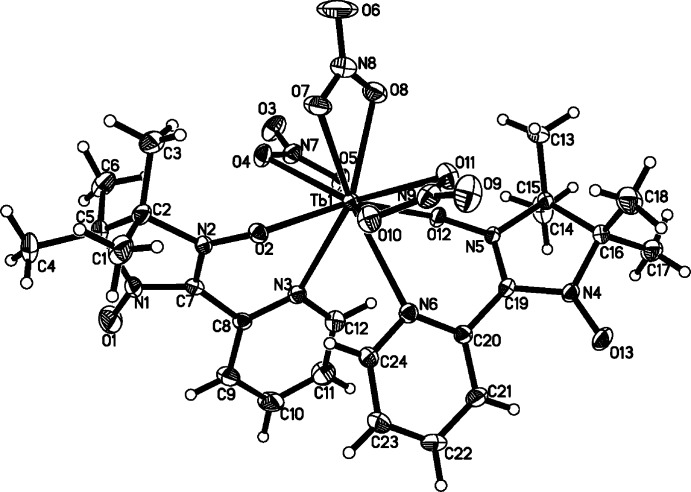
The molecular structure of the title compound drawn with 30% ellipsoidal probability.

**Fig. 2.**
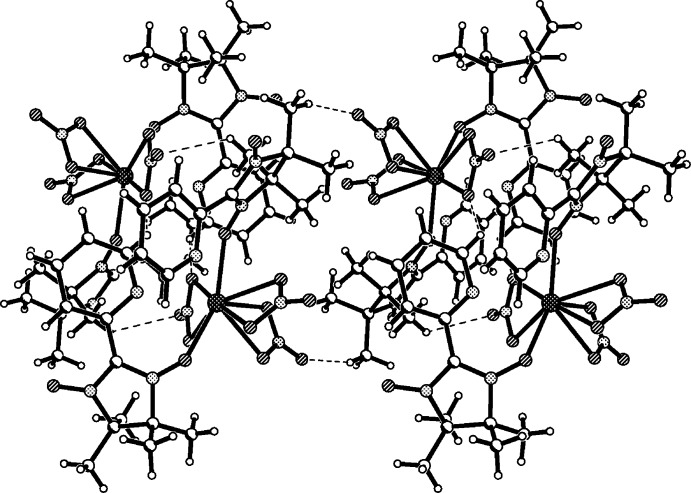
The three-dimensional framework of the structure connected through intermolecular hydrogen bonds.

## supplementary crystallographic information

### Comment

As a continuation of our work on complexes containing nitroxide radicals as the ligand, the title Tb complex is reported. An *ORTEP* drawing of [Tb^III^(NIT2Py)_2_(NO_3_)_3_] is illustrated in Fig. 1. The Tb^III^ ion is ten-coordinated by three η^2^-nitrato anions and two NIT2Py radicals which bind *via* one oxygen atom of the nitronyl nitroxide moiety and one nitrogen atom of the pyridine substituent. The complex is further connected by weak C—H···O H-bonds into a three-dimensional framework as shown in Fig. 2.

### Experimental

The compound was synthesized by the following procedure. Tb(NO_3_)_3_.6H_2_O (0.045 g, 0.2 mmol) and NIT2Py (0.047 g, 0.2 mmol) were dissolved in 10 mL of anhydrous THF. The mixture was stirred at room temperature for four hours and then filtered. The dark brown filtrate was allowed to stand in the dark for one week. Dark brown crystals were obtained.

### Refinement

Hydrogen atoms were placed in calculated positions (C—H = 0.93–0.96 Å) and refined using a riding model with *U_iso_*(H) = 1.2*U_eq_*(C_aromatic_) and 1.5*U_eq_*(C_methyl_).

### Crystal data

**Table:**

[Tb(NO_3_)_3_(C_12_H_16_N_3_O_2_)_2_]	*F*(000) = 1632
*M**_r_* = 813.51	*D*_x_ = 1.718 Mg m^−^^3^
Monoclinic, *P*2_1_/*n*	Mo *K*α radiation, λ = 0.71073 Å
Hall symbol: -P 2yn	Cell parameters from 9251 reflections
*a* = 12.292 (3) Å	θ = 3.0–27.9°
*b* = 11.114 (2) Å	µ = 2.33 mm^−^^1^
*c* = 23.264 (5) Å	*T* = 293 K
β = 98.37 (3)°	Prism, colorless
*V* = 3144.6 (11) Å^3^	0.20 × 0.20 × 0.20 mm
*Z* = 4	

### Data collection

**Table:**

Rigaku Saturn CCD area-detector diffractometer	5554 independent reflections
Radiation source: fine-focus sealed tube	4726 reflections with *I* > 2σ(*I*)
Graphite monochromator	*R*_int_ = 0.059
ω–θ scans	θ_max_ = 25.0°, θ_min_ = 2.0°
Absorption correction: multi-scan (*SADABS*; Sheldrick, 2004)	*h* = −14→14
*T*_min_ = 0.581, *T*_max_ = 1.000	*k* = −13→13
25443 measured reflections	*l* = −27→27

### Refinement

**Table:**

Refinement on *F*^2^	Primary atom site location: structure-invariant direct methods
Least-squares matrix: full	Secondary atom site location: difference Fourier map
*R*[*F*^2^ > 2σ(*F*^2^)] = 0.031	Hydrogen site location: inferred from neighbouring sites
*wR*(*F*^2^) = 0.071	H-atom parameters constrained
*S* = 1.01	*w* = 1/[σ^2^(*F*_o_^2^) + (0.0354*P*)^2^] where *P* = (*F*_o_^2^ + 2*F*_c_^2^)/3
5554 reflections	(Δ/σ)_max_ = 0.001
432 parameters	Δρ_max_ = 0.89 e Å^−^^3^
0 restraints	Δρ_min_ = −0.95 e Å^−^^3^

### Special details

**Table:**

Geometry. All esds (except the esd in the dihedral angle between two l.s. planes) are estimated using the full covariance matrix. The cell esds are taken into account individually in the estimation of esds in distances, angles and torsion angles; correlations between esds in cell parameters are only used when they are defined by crystal symmetry. An approximate (isotropic) treatment of cell esds is used for estimating esds involving l.s. planes.
Refinement. Refinement of *F*^2^ against ALL reflections. The weighted *R*-factor *wR* and goodness of fit *S* are based on *F*^2^, conventional *R*-factors *R* are based on *F*, with *F* set to zero for negative *F*^2^. The threshold expression of *F*^2^ > σ(*F*^2^) is used only for calculating *R*-factors(gt) *etc*. and is not relevant to the choice of reflections for refinement. *R*-factors based on *F*^2^ are statistically about twice as large as those based on *F*, and *R*- factors based on ALL data will be even larger.

### Fractional atomic coordinates and isotropic or equivalent isotropic displacement parameters (Å^2^)

**Table:**

	*x*	*y*	*z*	*U*_iso_*/*U*_eq_	
Tb1	0.179699 (13)	0.838779 (16)	0.103896 (7)	0.02337 (7)	
C1	0.5499 (3)	1.0194 (4)	0.21909 (17)	0.0512 (12)	
H1A	0.5285	1.0905	0.2381	0.077*	
H1B	0.6286	1.0143	0.2239	0.077*	
H1C	0.5209	0.9497	0.2360	0.077*	
C2	0.5046 (3)	1.0251 (4)	0.15437 (16)	0.0342 (9)	
C3	0.5415 (3)	0.9164 (4)	0.12280 (18)	0.0470 (11)	
H3A	0.5285	0.8446	0.1437	0.071*	
H3B	0.6186	0.9232	0.1204	0.071*	
H3C	0.5009	0.9127	0.0843	0.071*	
C4	0.6215 (3)	1.2196 (5)	0.1523 (2)	0.0620 (14)	
H4A	0.6208	1.2973	0.1342	0.093*	
H4B	0.6869	1.1771	0.1462	0.093*	
H4C	0.6204	1.2292	0.1932	0.093*	
C5	0.5205 (3)	1.1483 (4)	0.12557 (18)	0.0366 (10)	
C6	0.5140 (3)	1.1439 (4)	0.05979 (18)	0.0506 (12)	
H6A	0.4485	1.1020	0.0434	0.076*	
H6B	0.5772	1.1026	0.0498	0.076*	
H6C	0.5122	1.2244	0.0447	0.076*	
C7	0.3375 (3)	1.1328 (3)	0.14343 (15)	0.0273 (8)	
C8	0.2205 (3)	1.1605 (3)	0.13872 (15)	0.0264 (8)	
C9	0.1869 (3)	1.2733 (4)	0.15514 (17)	0.0392 (10)	
H9	0.2375	1.3287	0.1731	0.047*	
C10	0.0755 (3)	1.3009 (4)	0.1439 (2)	0.0516 (12)	
H10	0.0498	1.3752	0.1546	0.062*	
C11	0.0038 (4)	1.2166 (4)	0.11684 (19)	0.0496 (12)	
H11	−0.0707	1.2341	0.1075	0.060*	
C12	0.0440 (3)	1.1056 (4)	0.10369 (16)	0.0342 (9)	
H12	−0.0054	1.0489	0.0858	0.041*	
C13	−0.0898 (3)	0.5854 (4)	0.02645 (19)	0.0527 (13)	
H13A	−0.0490	0.6257	0.0000	0.079*	
H13B	−0.1357	0.5247	0.0060	0.079*	
H13C	−0.0397	0.5485	0.0569	0.079*	
C14	−0.2299 (3)	0.7483 (4)	0.00525 (17)	0.0502 (12)	
H14A	−0.2748	0.8043	0.0227	0.075*	
H14B	−0.2760	0.6949	−0.0200	0.075*	
H14C	−0.1824	0.7915	−0.0168	0.075*	
C15	−0.1608 (3)	0.6758 (3)	0.05252 (15)	0.0276 (8)	
C16	−0.2276 (3)	0.6246 (3)	0.09918 (16)	0.0282 (8)	
C17	−0.3508 (3)	0.6170 (4)	0.08142 (19)	0.0464 (11)	
H17A	−0.3841	0.5853	0.1131	0.070*	
H17B	−0.3668	0.5649	0.0483	0.070*	
H17C	−0.3795	0.6958	0.0716	0.070*	
C18	−0.1832 (4)	0.5038 (4)	0.12504 (19)	0.0554 (12)	
H18A	−0.1054	0.5102	0.1373	0.083*	
H18B	−0.1974	0.4420	0.0961	0.083*	
H18C	−0.2191	0.4840	0.1578	0.083*	
C19	−0.1140 (3)	0.7868 (3)	0.13971 (14)	0.0248 (8)	
C20	−0.0612 (3)	0.8733 (3)	0.18217 (15)	0.0267 (8)	
C21	−0.1226 (3)	0.9372 (4)	0.21670 (16)	0.0403 (10)	
H21	−0.1983	0.9263	0.2131	0.048*	
C22	−0.0699 (3)	1.0181 (4)	0.25706 (17)	0.0482 (12)	
H22	−0.1099	1.0657	0.2794	0.058*	
C23	0.0425 (3)	1.0260 (4)	0.26313 (17)	0.0444 (11)	
H23	0.0809	1.0750	0.2915	0.053*	
C24	0.0971 (3)	0.9599 (3)	0.22643 (15)	0.0325 (9)	
H24	0.1732	0.9670	0.2306	0.039*	
N1	0.4181 (2)	1.2129 (3)	0.13688 (14)	0.0360 (8)	
N2	0.3810 (2)	1.0241 (3)	0.15072 (12)	0.0269 (7)	
N3	0.1495 (2)	1.0751 (3)	0.11510 (12)	0.0269 (7)	
N4	−0.2016 (2)	0.7157 (3)	0.14721 (12)	0.0272 (7)	
N5	−0.0869 (2)	0.7638 (3)	0.08801 (11)	0.0224 (6)	
N6	0.0488 (2)	0.8867 (3)	0.18532 (12)	0.0281 (7)	
N7	0.2135 (2)	0.9484 (3)	−0.00603 (13)	0.0334 (8)	
N8	0.2955 (3)	0.6582 (3)	0.04797 (18)	0.0544 (11)	
N9	0.1719 (3)	0.6418 (3)	0.18668 (15)	0.0360 (8)	
O1	0.4056 (3)	1.3255 (3)	0.12888 (16)	0.0603 (9)	
O2	0.33022 (18)	0.9255 (2)	0.16160 (10)	0.0279 (6)	
O3	0.2273 (2)	0.9783 (3)	−0.05526 (11)	0.0507 (8)	
O4	0.29363 (19)	0.9350 (2)	0.03460 (11)	0.0364 (7)	
O5	0.11923 (18)	0.9293 (3)	0.00678 (10)	0.0352 (6)	
O6	0.3472 (3)	0.5873 (4)	0.02285 (18)	0.1058 (16)	
O7	0.3335 (2)	0.7016 (3)	0.09700 (13)	0.0468 (8)	
O8	0.1999 (2)	0.6941 (3)	0.02583 (13)	0.0475 (8)	
O9	0.1640 (2)	0.5585 (3)	0.22000 (13)	0.0593 (9)	
O10	0.2374 (2)	0.7298 (3)	0.19933 (11)	0.0395 (7)	
O11	0.1141 (2)	0.6446 (2)	0.13669 (12)	0.0393 (7)	
O12	−0.00934 (19)	0.8177 (2)	0.06566 (10)	0.0284 (6)	
O13	−0.2481 (2)	0.7132 (3)	0.19264 (11)	0.0446 (7)	

### Atomic displacement parameters (Å^2^)

**Table:**

	*U*^11^	*U*^22^	*U*^33^	*U*^12^	*U*^13^	*U*^23^
Tb1	0.02218 (11)	0.02498 (12)	0.02300 (11)	−0.00103 (8)	0.00341 (7)	−0.00178 (7)
C1	0.027 (2)	0.080 (4)	0.044 (3)	0.002 (2)	−0.0055 (19)	−0.003 (2)
C2	0.0182 (18)	0.048 (3)	0.036 (2)	0.0018 (18)	0.0017 (16)	−0.0084 (19)
C3	0.035 (2)	0.048 (3)	0.058 (3)	0.009 (2)	0.008 (2)	−0.009 (2)
C4	0.032 (2)	0.064 (4)	0.089 (4)	−0.021 (2)	0.008 (2)	−0.014 (3)
C5	0.024 (2)	0.039 (3)	0.046 (2)	−0.0066 (18)	0.0044 (18)	−0.0090 (19)
C6	0.037 (2)	0.072 (4)	0.046 (3)	0.002 (2)	0.015 (2)	0.003 (2)
C7	0.029 (2)	0.025 (2)	0.0274 (19)	0.0000 (16)	0.0047 (16)	−0.0067 (16)
C8	0.0275 (19)	0.030 (2)	0.0213 (18)	0.0013 (17)	0.0017 (15)	−0.0027 (16)
C9	0.039 (2)	0.036 (3)	0.041 (2)	0.006 (2)	−0.0012 (19)	−0.0101 (19)
C10	0.044 (3)	0.042 (3)	0.068 (3)	0.017 (2)	0.006 (2)	−0.016 (2)
C11	0.037 (2)	0.048 (3)	0.063 (3)	0.011 (2)	0.002 (2)	0.000 (2)
C12	0.032 (2)	0.035 (2)	0.035 (2)	0.0023 (19)	0.0012 (18)	0.0041 (18)
C13	0.055 (3)	0.047 (3)	0.062 (3)	−0.014 (2)	0.029 (2)	−0.029 (2)
C14	0.052 (3)	0.058 (3)	0.036 (2)	−0.016 (2)	−0.009 (2)	0.011 (2)
C15	0.0285 (19)	0.027 (2)	0.028 (2)	−0.0079 (16)	0.0075 (16)	−0.0056 (16)
C16	0.0277 (19)	0.026 (2)	0.032 (2)	−0.0048 (16)	0.0066 (16)	−0.0013 (16)
C17	0.031 (2)	0.060 (3)	0.049 (3)	−0.008 (2)	0.008 (2)	−0.017 (2)
C18	0.075 (3)	0.037 (3)	0.056 (3)	−0.001 (2)	0.017 (3)	0.009 (2)
C19	0.0203 (18)	0.028 (2)	0.0268 (19)	−0.0005 (16)	0.0058 (15)	0.0018 (16)
C20	0.0258 (19)	0.027 (2)	0.0267 (19)	−0.0001 (16)	0.0033 (16)	−0.0018 (16)
C21	0.029 (2)	0.053 (3)	0.041 (2)	−0.001 (2)	0.0114 (18)	−0.013 (2)
C22	0.050 (3)	0.052 (3)	0.046 (3)	0.000 (2)	0.018 (2)	−0.022 (2)
C23	0.052 (3)	0.046 (3)	0.036 (2)	−0.011 (2)	0.008 (2)	−0.016 (2)
C24	0.030 (2)	0.037 (2)	0.030 (2)	−0.0058 (18)	0.0021 (17)	−0.0068 (18)
N1	0.0300 (17)	0.0256 (19)	0.053 (2)	−0.0054 (15)	0.0079 (16)	−0.0078 (16)
N2	0.0204 (15)	0.033 (2)	0.0263 (16)	−0.0030 (14)	0.0013 (13)	−0.0074 (14)
N3	0.0245 (15)	0.0304 (19)	0.0256 (16)	0.0010 (14)	0.0025 (13)	−0.0008 (14)
N4	0.0262 (16)	0.0329 (19)	0.0233 (16)	−0.0043 (14)	0.0068 (13)	−0.0005 (14)
N5	0.0195 (14)	0.0236 (17)	0.0245 (15)	−0.0018 (13)	0.0046 (12)	0.0007 (13)
N6	0.0255 (16)	0.0309 (18)	0.0274 (16)	−0.0010 (14)	0.0025 (13)	−0.0003 (14)
N7	0.0307 (18)	0.041 (2)	0.0296 (18)	−0.0005 (15)	0.0072 (15)	0.0003 (15)
N8	0.054 (3)	0.044 (3)	0.068 (3)	0.000 (2)	0.019 (2)	−0.018 (2)
N9	0.0303 (18)	0.038 (2)	0.041 (2)	0.0093 (16)	0.0062 (16)	0.0095 (17)
O1	0.057 (2)	0.0310 (19)	0.095 (3)	−0.0057 (15)	0.0164 (19)	−0.0009 (17)
O2	0.0255 (13)	0.0275 (15)	0.0287 (13)	0.0003 (12)	−0.0025 (11)	−0.0024 (11)
O3	0.0464 (17)	0.078 (2)	0.0306 (15)	−0.0007 (16)	0.0160 (13)	0.0132 (15)
O4	0.0244 (13)	0.0506 (19)	0.0336 (15)	−0.0066 (13)	0.0023 (12)	−0.0005 (13)
O5	0.0211 (13)	0.0544 (19)	0.0299 (14)	−0.0065 (13)	0.0032 (11)	0.0036 (13)
O6	0.086 (3)	0.106 (4)	0.126 (4)	0.028 (3)	0.020 (3)	−0.078 (3)
O7	0.0440 (17)	0.0422 (18)	0.0526 (19)	0.0099 (14)	0.0009 (15)	−0.0186 (15)
O8	0.0453 (18)	0.0449 (19)	0.0511 (19)	−0.0009 (15)	0.0031 (15)	−0.0186 (15)
O9	0.060 (2)	0.056 (2)	0.061 (2)	−0.0014 (17)	0.0051 (16)	0.0338 (18)
O10	0.0340 (15)	0.0411 (19)	0.0404 (16)	0.0008 (14)	−0.0043 (13)	0.0035 (14)
O11	0.0405 (16)	0.0327 (17)	0.0417 (17)	−0.0021 (13)	−0.0035 (14)	0.0055 (13)
O12	0.0236 (13)	0.0353 (16)	0.0272 (13)	−0.0062 (11)	0.0071 (11)	0.0011 (11)
O13	0.0435 (16)	0.061 (2)	0.0340 (16)	−0.0152 (15)	0.0210 (13)	−0.0046 (14)

### Geometric parameters (Å, º)

**Table:**

Tb1—O2	2.331 (2)	C13—H13B	0.9600
Tb1—O12	2.376 (2)	C13—H13C	0.9600
Tb1—O7	2.452 (3)	C14—C15	1.519 (5)
Tb1—O11	2.463 (3)	C14—H14A	0.9600
Tb1—O8	2.465 (3)	C14—H14B	0.9600
Tb1—O5	2.486 (2)	C14—H14C	0.9600
Tb1—O4	2.522 (2)	C15—N5	1.499 (4)
Tb1—O10	2.538 (3)	C15—C16	1.561 (5)
Tb1—N3	2.671 (3)	C16—N4	1.507 (5)
Tb1—N6	2.711 (3)	C16—C17	1.513 (5)
C1—C2	1.529 (5)	C16—C18	1.538 (5)
C1—H1A	0.9600	C17—H17A	0.9600
C1—H1B	0.9600	C17—H17B	0.9600
C1—H1C	0.9600	C17—H17C	0.9600
C2—N2	1.509 (4)	C18—H18A	0.9600
C2—C3	1.518 (5)	C18—H18B	0.9600
C2—C5	1.549 (5)	C18—H18C	0.9600
C3—H3A	0.9600	C19—N5	1.319 (4)
C3—H3B	0.9600	C19—N4	1.367 (4)
C3—H3C	0.9600	C19—C20	1.461 (5)
C4—C5	1.527 (5)	C20—N6	1.352 (4)
C4—H4A	0.9600	C20—C21	1.376 (5)
C4—H4B	0.9600	C21—C22	1.390 (5)
C4—H4C	0.9600	C21—H21	0.9300
C5—N1	1.505 (5)	C22—C23	1.371 (5)
C5—C6	1.521 (5)	C22—H22	0.9300
C6—H6A	0.9600	C23—C24	1.373 (5)
C6—H6B	0.9600	C23—H23	0.9300
C6—H6C	0.9600	C24—N6	1.328 (4)
C7—N2	1.321 (4)	C24—H24	0.9300
C7—N1	1.358 (5)	N1—O1	1.271 (4)
C7—C8	1.459 (5)	N2—O2	1.305 (4)
C8—N3	1.351 (4)	N4—O13	1.273 (3)
C8—C9	1.391 (5)	N5—O12	1.297 (3)
C9—C10	1.392 (5)	N7—O3	1.228 (4)
C9—H9	0.9300	N7—O5	1.256 (3)
C10—C11	1.375 (6)	N7—O4	1.271 (4)
C10—H10	0.9300	N8—O6	1.214 (4)
C11—C12	1.380 (6)	N8—O7	1.263 (5)
C11—H11	0.9300	N8—O8	1.277 (5)
C12—N3	1.329 (4)	N9—O9	1.220 (4)
C12—H12	0.9300	N9—O11	1.272 (4)
C13—C15	1.514 (5)	N9—O10	1.274 (4)
C13—H13A	0.9600		
			
O2—Tb1—O12	155.19 (8)	C10—C11—H11	120.5
O2—Tb1—O7	74.92 (9)	C12—C11—H11	120.5
O12—Tb1—O7	129.47 (9)	N3—C12—C11	123.7 (4)
O2—Tb1—O11	116.88 (9)	N3—C12—H12	118.2
O12—Tb1—O11	71.54 (9)	C11—C12—H12	118.2
O7—Tb1—O11	76.45 (10)	C15—C13—H13A	109.5
O2—Tb1—O8	122.52 (9)	C15—C13—H13B	109.5
O12—Tb1—O8	81.82 (9)	H13A—C13—H13B	109.5
O7—Tb1—O8	52.04 (10)	C15—C13—H13C	109.5
O11—Tb1—O8	74.29 (10)	H13A—C13—H13C	109.5
O2—Tb1—O5	117.61 (8)	H13B—C13—H13C	109.5
O12—Tb1—O5	63.44 (8)	C15—C14—H14A	109.5
O7—Tb1—O5	108.89 (9)	C15—C14—H14B	109.5
O11—Tb1—O5	124.61 (9)	H14A—C14—H14B	109.5
O8—Tb1—O5	69.11 (10)	C15—C14—H14C	109.5
O2—Tb1—O4	74.03 (8)	H14A—C14—H14C	109.5
O12—Tb1—O4	113.97 (8)	H14B—C14—H14C	109.5
O7—Tb1—O4	73.55 (10)	N5—C15—C13	108.4 (3)
O11—Tb1—O4	143.77 (9)	N5—C15—C14	106.4 (3)
O8—Tb1—O4	71.39 (9)	C13—C15—C14	110.8 (3)
O5—Tb1—O4	50.79 (8)	N5—C15—C16	101.1 (3)
O2—Tb1—O10	66.18 (9)	C13—C15—C16	115.4 (3)
O12—Tb1—O10	114.46 (8)	C14—C15—C16	113.7 (3)
O7—Tb1—O10	68.94 (10)	N4—C16—C17	109.6 (3)
O11—Tb1—O10	51.20 (9)	N4—C16—C18	105.8 (3)
O8—Tb1—O10	106.73 (10)	C17—C16—C18	110.0 (3)
O5—Tb1—O10	175.35 (9)	N4—C16—C15	101.2 (3)
O4—Tb1—O10	130.63 (8)	C17—C16—C15	115.9 (3)
O2—Tb1—N3	69.33 (8)	C18—C16—C15	113.5 (3)
O12—Tb1—N3	89.49 (8)	C16—C17—H17A	109.5
O7—Tb1—N3	137.51 (9)	C16—C17—H17B	109.5
O11—Tb1—N3	140.80 (9)	H17A—C17—H17B	109.5
O8—Tb1—N3	138.05 (10)	C16—C17—H17C	109.5
O5—Tb1—N3	70.31 (9)	H17A—C17—H17C	109.5
O4—Tb1—N3	75.13 (9)	H17B—C17—H17C	109.5
O10—Tb1—N3	114.14 (9)	C16—C18—H18A	109.5
O2—Tb1—N6	91.06 (8)	C16—C18—H18B	109.5
O12—Tb1—N6	68.48 (8)	H18A—C18—H18B	109.5
O7—Tb1—N6	135.20 (10)	C16—C18—H18C	109.5
O11—Tb1—N6	72.62 (9)	H18A—C18—H18C	109.5
O8—Tb1—N6	140.94 (10)	H18B—C18—H18C	109.5
O5—Tb1—N6	115.22 (8)	N5—C19—N4	108.2 (3)
O4—Tb1—N6	143.59 (9)	N5—C19—C20	126.7 (3)
O10—Tb1—N6	66.49 (9)	N4—C19—C20	125.1 (3)
N3—Tb1—N6	68.51 (9)	N6—C20—C21	122.7 (3)
C2—C1—H1A	109.5	N6—C20—C19	116.8 (3)
C2—C1—H1B	109.5	C21—C20—C19	120.5 (3)
H1A—C1—H1B	109.5	C20—C21—C22	119.1 (4)
C2—C1—H1C	109.5	C20—C21—H21	120.4
H1A—C1—H1C	109.5	C22—C21—H21	120.4
H1B—C1—H1C	109.5	C23—C22—C21	118.3 (4)
N2—C2—C3	109.7 (3)	C23—C22—H22	120.8
N2—C2—C1	105.9 (3)	C21—C22—H22	120.8
C3—C2—C1	110.6 (3)	C22—C23—C24	118.6 (4)
N2—C2—C5	99.9 (3)	C22—C23—H23	120.7
C3—C2—C5	115.4 (3)	C24—C23—H23	120.7
C1—C2—C5	114.4 (3)	N6—C24—C23	124.6 (4)
C2—C3—H3A	109.5	N6—C24—H24	117.7
C2—C3—H3B	109.5	C23—C24—H24	117.7
H3A—C3—H3B	109.5	O1—N1—C7	126.0 (3)
C2—C3—H3C	109.5	O1—N1—C5	122.1 (3)
H3A—C3—H3C	109.5	C7—N1—C5	110.4 (3)
H3B—C3—H3C	109.5	O2—N2—C7	126.6 (3)
C5—C4—H4A	109.5	O2—N2—C2	120.1 (3)
C5—C4—H4B	109.5	C7—N2—C2	112.7 (3)
H4A—C4—H4B	109.5	C12—N3—C8	117.2 (3)
C5—C4—H4C	109.5	C12—N3—Tb1	112.1 (3)
H4A—C4—H4C	109.5	C8—N3—Tb1	129.8 (2)
H4B—C4—H4C	109.5	O13—N4—C19	125.1 (3)
N1—C5—C6	105.6 (3)	O13—N4—C16	121.9 (3)
N1—C5—C4	109.4 (3)	C19—N4—C16	112.3 (3)
C6—C5—C4	110.2 (3)	O12—N5—C19	125.1 (3)
N1—C5—C2	100.6 (3)	O12—N5—C15	120.2 (3)
C6—C5—C2	114.6 (3)	C19—N5—C15	114.5 (3)
C4—C5—C2	115.4 (4)	C24—N6—C20	116.4 (3)
C5—C6—H6A	109.5	C24—N6—Tb1	112.0 (2)
C5—C6—H6B	109.5	C20—N6—Tb1	129.2 (2)
H6A—C6—H6B	109.5	O3—N7—O5	121.6 (3)
C5—C6—H6C	109.5	O3—N7—O4	121.9 (3)
H6A—C6—H6C	109.5	O5—N7—O4	116.5 (3)
H6B—C6—H6C	109.5	O6—N8—O7	122.1 (4)
N2—C7—N1	109.0 (3)	O6—N8—O8	121.6 (4)
N2—C7—C8	125.4 (3)	O7—N8—O8	116.3 (3)
N1—C7—C8	125.5 (3)	O9—N9—O11	121.0 (4)
N3—C8—C9	123.0 (3)	O9—N9—O10	122.8 (4)
N3—C8—C7	117.0 (3)	O11—N9—O10	116.2 (3)
C9—C8—C7	119.9 (3)	N2—O2—Tb1	126.72 (19)
C8—C9—C10	118.1 (4)	N7—O4—Tb1	94.75 (18)
C8—C9—H9	120.9	N7—O5—Tb1	96.88 (19)
C10—C9—H9	120.9	N8—O7—Tb1	96.3 (2)
C11—C10—C9	118.9 (4)	N8—O8—Tb1	95.3 (2)
C11—C10—H10	120.5	N9—O10—Tb1	94.5 (2)
C9—C10—H10	120.5	N9—O11—Tb1	98.1 (2)
C10—C11—C12	119.0 (4)	N5—O12—Tb1	129.14 (19)
			
N2—C2—C5—N1	24.8 (3)	C19—C20—N6—Tb1	23.2 (5)
C3—C2—C5—N1	142.3 (3)	O2—Tb1—N6—C24	11.2 (3)
C1—C2—C5—N1	−87.8 (4)	O12—Tb1—N6—C24	−154.4 (3)
N2—C2—C5—C6	−87.9 (3)	O7—Tb1—N6—C24	80.7 (3)
C3—C2—C5—C6	29.6 (5)	O11—Tb1—N6—C24	129.1 (3)
C1—C2—C5—C6	159.5 (3)	O8—Tb1—N6—C24	162.4 (2)
N2—C2—C5—C4	142.4 (3)	O5—Tb1—N6—C24	−110.2 (3)
C3—C2—C5—C4	−100.1 (4)	O4—Tb1—N6—C24	−52.8 (3)
C1—C2—C5—C4	29.8 (5)	O10—Tb1—N6—C24	74.6 (3)
N2—C7—C8—N3	−30.5 (5)	N3—Tb1—N6—C24	−56.0 (3)
N1—C7—C8—N3	144.5 (4)	O2—Tb1—N6—C20	172.7 (3)
N2—C7—C8—C9	153.1 (4)	O12—Tb1—N6—C20	7.1 (3)
N1—C7—C8—C9	−31.9 (6)	O7—Tb1—N6—C20	−117.8 (3)
N3—C8—C9—C10	−3.3 (6)	O11—Tb1—N6—C20	−69.4 (3)
C7—C8—C9—C10	172.9 (4)	O8—Tb1—N6—C20	−36.1 (4)
C8—C9—C10—C11	−0.6 (7)	O5—Tb1—N6—C20	51.3 (3)
C9—C10—C11—C12	2.5 (7)	O4—Tb1—N6—C20	108.7 (3)
C10—C11—C12—N3	−0.9 (7)	O10—Tb1—N6—C20	−123.9 (3)
N5—C15—C16—N4	14.8 (3)	N3—Tb1—N6—C20	105.5 (3)
C13—C15—C16—N4	131.5 (3)	C7—N2—O2—Tb1	59.6 (4)
C14—C15—C16—N4	−98.8 (3)	C2—N2—O2—Tb1	−129.8 (3)
N5—C15—C16—C17	133.3 (3)	O12—Tb1—O2—N2	−79.2 (3)
C13—C15—C16—C17	−110.0 (4)	O7—Tb1—O2—N2	110.4 (2)
C14—C15—C16—C17	19.7 (5)	O11—Tb1—O2—N2	176.3 (2)
N5—C15—C16—C18	−98.0 (3)	O8—Tb1—O2—N2	88.4 (3)
C13—C15—C16—C18	18.7 (5)	O5—Tb1—O2—N2	6.7 (3)
C14—C15—C16—C18	148.3 (4)	O4—Tb1—O2—N2	33.6 (2)
N5—C19—C20—N6	−35.5 (6)	O10—Tb1—O2—N2	−176.3 (3)
N4—C19—C20—N6	144.9 (3)	N3—Tb1—O2—N2	−46.2 (2)
N5—C19—C20—C21	144.4 (4)	N6—Tb1—O2—N2	−112.7 (2)
N4—C19—C20—C21	−35.2 (6)	O3—N7—O4—Tb1	−169.6 (3)
N6—C20—C21—C22	−0.7 (6)	O5—N7—O4—Tb1	10.4 (3)
C19—C20—C21—C22	179.4 (4)	O2—Tb1—O4—N7	−154.9 (2)
C20—C21—C22—C23	−3.7 (7)	O12—Tb1—O4—N7	0.1 (2)
C21—C22—C23—C24	4.4 (7)	O7—Tb1—O4—N7	126.5 (2)
C22—C23—C24—N6	−0.9 (7)	O11—Tb1—O4—N7	91.3 (2)
N2—C7—N1—O1	179.9 (4)	O8—Tb1—O4—N7	71.8 (2)
C8—C7—N1—O1	4.2 (6)	O5—Tb1—O4—N7	−6.03 (18)
N2—C7—N1—C5	13.8 (4)	O10—Tb1—O4—N7	168.19 (18)
C8—C7—N1—C5	−161.8 (3)	N3—Tb1—O4—N7	−82.5 (2)
C6—C5—N1—O1	−72.5 (4)	N6—Tb1—O4—N7	−85.7 (2)
C4—C5—N1—O1	46.2 (5)	O3—N7—O5—Tb1	169.4 (3)
C2—C5—N1—O1	168.1 (4)	O4—N7—O5—Tb1	−10.6 (3)
C6—C5—N1—C7	94.2 (4)	O2—Tb1—O5—N7	40.3 (2)
C4—C5—N1—C7	−147.2 (4)	O12—Tb1—O5—N7	−167.6 (2)
C2—C5—N1—C7	−25.2 (4)	O7—Tb1—O5—N7	−42.2 (2)
N1—C7—N2—O2	175.9 (3)	O11—Tb1—O5—N7	−128.4 (2)
C8—C7—N2—O2	−8.4 (6)	O8—Tb1—O5—N7	−76.4 (2)
N1—C7—N2—C2	4.7 (4)	O4—Tb1—O5—N7	6.13 (19)
C8—C7—N2—C2	−179.6 (3)	N3—Tb1—O5—N7	92.7 (2)
C3—C2—N2—O2	46.7 (4)	N6—Tb1—O5—N7	145.9 (2)
C1—C2—N2—O2	−72.7 (4)	O6—N8—O7—Tb1	175.6 (4)
C5—C2—N2—O2	168.3 (3)	O8—N8—O7—Tb1	−3.7 (4)
C3—C2—N2—C7	−141.5 (3)	O2—Tb1—O7—N8	−154.2 (3)
C1—C2—N2—C7	99.2 (4)	O12—Tb1—O7—N8	31.0 (3)
C5—C2—N2—C7	−19.8 (4)	O11—Tb1—O7—N8	82.7 (2)
C11—C12—N3—C8	−2.8 (5)	O8—Tb1—O7—N8	2.2 (2)
C11—C12—N3—Tb1	167.1 (3)	O5—Tb1—O7—N8	−39.7 (3)
C9—C8—N3—C12	4.9 (5)	O4—Tb1—O7—N8	−76.8 (2)
C7—C8—N3—C12	−171.4 (3)	O10—Tb1—O7—N8	135.9 (3)
C9—C8—N3—Tb1	−162.9 (3)	N3—Tb1—O7—N8	−120.9 (2)
C7—C8—N3—Tb1	20.9 (4)	N6—Tb1—O7—N8	129.9 (2)
O2—Tb1—N3—C12	−159.6 (3)	O6—N8—O8—Tb1	−175.6 (4)
O12—Tb1—N3—C12	7.2 (2)	O7—N8—O8—Tb1	3.7 (4)
O7—Tb1—N3—C12	165.9 (2)	O2—Tb1—O8—N8	25.2 (3)
O11—Tb1—N3—C12	−52.0 (3)	O12—Tb1—O8—N8	−160.0 (3)
O8—Tb1—N3—C12	84.3 (3)	O7—Tb1—O8—N8	−2.1 (2)
O5—Tb1—N3—C12	69.1 (2)	O11—Tb1—O8—N8	−87.0 (2)
O4—Tb1—N3—C12	122.2 (2)	O5—Tb1—O8—N8	135.3 (3)
O10—Tb1—N3—C12	−109.5 (2)	O4—Tb1—O8—N8	81.2 (2)
N6—Tb1—N3—C12	−59.8 (2)	O10—Tb1—O8—N8	−46.9 (3)
O2—Tb1—N3—C8	8.7 (3)	N3—Tb1—O8—N8	119.9 (2)
O12—Tb1—N3—C8	175.4 (3)	N6—Tb1—O8—N8	−120.0 (2)
O7—Tb1—N3—C8	−25.9 (3)	O9—N9—O10—Tb1	−178.7 (3)
O11—Tb1—N3—C8	116.2 (3)	O11—N9—O10—Tb1	1.4 (3)
O8—Tb1—N3—C8	−107.4 (3)	O2—Tb1—O10—N9	−172.4 (2)
O5—Tb1—N3—C8	−122.7 (3)	O12—Tb1—O10—N9	34.8 (2)
O4—Tb1—N3—C8	−69.5 (3)	O7—Tb1—O10—N9	−90.1 (2)
O10—Tb1—N3—C8	58.7 (3)	O11—Tb1—O10—N9	−0.85 (18)
N6—Tb1—N3—C8	108.5 (3)	O8—Tb1—O10—N9	−53.7 (2)
N5—C19—N4—O13	178.9 (3)	N3—Tb1—O10—N9	135.97 (19)
C20—C19—N4—O13	−1.5 (6)	N6—Tb1—O10—N9	85.2 (2)
N5—C19—N4—C16	7.9 (4)	O9—N9—O11—Tb1	178.6 (3)
C20—C19—N4—C16	−172.4 (3)	O10—N9—O11—Tb1	−1.5 (3)
C17—C16—N4—O13	50.9 (5)	O2—Tb1—O11—N9	9.5 (2)
C18—C16—N4—O13	−67.6 (4)	O12—Tb1—O11—N9	−145.1 (2)
C15—C16—N4—O13	173.8 (3)	O7—Tb1—O11—N9	74.6 (2)
C17—C16—N4—C19	−137.8 (3)	O8—Tb1—O11—N9	128.4 (2)
C18—C16—N4—C19	103.7 (3)	O5—Tb1—O11—N9	178.35 (18)
C15—C16—N4—C19	−14.9 (4)	O4—Tb1—O11—N9	109.3 (2)
N4—C19—N5—O12	177.7 (3)	O10—Tb1—O11—N9	0.86 (18)
C20—C19—N5—O12	−1.9 (6)	N3—Tb1—O11—N9	−80.2 (2)
N4—C19—N5—C15	3.4 (4)	N6—Tb1—O11—N9	−72.6 (2)
C20—C19—N5—C15	−176.3 (3)	C19—N5—O12—Tb1	55.5 (4)
C13—C15—N5—O12	51.4 (4)	C15—N5—O12—Tb1	−130.4 (3)
C14—C15—N5—O12	−67.9 (4)	O2—Tb1—O12—N5	−82.1 (3)
C16—C15—N5—O12	173.1 (3)	O7—Tb1—O12—N5	85.8 (3)
C13—C15—N5—C19	−134.0 (3)	O11—Tb1—O12—N5	32.3 (2)
C14—C15—N5—C19	106.7 (4)	O8—Tb1—O12—N5	108.4 (3)
C16—C15—N5—C19	−12.3 (4)	O5—Tb1—O12—N5	179.1 (3)
C23—C24—N6—C20	−3.4 (6)	O4—Tb1—O12—N5	173.8 (2)
C23—C24—N6—Tb1	160.7 (3)	O10—Tb1—O12—N5	3.7 (3)
C21—C20—N6—C24	4.1 (5)	N3—Tb1—O12—N5	−112.8 (3)
C19—C20—N6—C24	−176.0 (3)	N6—Tb1—O12—N5	−45.8 (2)
C21—C20—N6—Tb1	−156.7 (3)		

### Hydrogen-bond geometry (Å, º)

**Table:**

*D*—H···*A*	*D*—H	H···*A*	*D*···*A*	*D*—H···*A*
C22—H22···O13^i^	0.93	2.55	3.440 (5)	161
C17—H17*B*···O6^ii^	0.96	2.40	3.327 (5)	161
C6—H6*B*···O3^iii^	0.96	2.55	3.473 (5)	161
C24—H24···O9^iv^	0.93	2.38	3.211 (5)	148

Symmetry codes: (i) −*x*−1/2, *y*+1/2, −*z*+1/2; (ii) −*x*, −*y*+1, −*z*; (iii) −*x*+1, −*y*+2, −*z*; (iv) −*x*+1/2, *y*+1/2, −*z*+1/2.
